# Analysis of the Relationship Between Personal Characteristics and Alcohol Consumption Behavior of Chinese Consumers

**DOI:** 10.3390/foods14203536

**Published:** 2025-10-17

**Authors:** Xin Yuan, Yiyuan Chen, Ruiyang Yin, Liyun Guo, Yumei Song, Bofeng Zhong, Dongrui Zhao

**Affiliations:** 1 China Food Flavor and Nutrition Health Innovation Center, Beijing Technology and Business University, Beijing 100048, China; yuanxin5215@163.com (X.Y.); chenyiyuan_1112@163.com (Y.C.); z125678hhh@outlook.com (B.Z.); 2 Beijing Laboratory of Food Quality and Safety, Beijing Technology and Business University, Beijing 100048, China; 3 Key Laboratory of Brewing Molecular Engineering of China Light Industry, Beijing Technology & Business University, Beijing 100048, China; 4 School of Food and Health, Beijing Technology and Business University, Beijing 100048, China; 5 Technology Center of Beijing Yanjing Beer Co., Ltd., Beijing 101300, China; ruiyang_yin@163.com (R.Y.); yanjing6089@163.com (L.G.); songym@163.com (Y.S.)

**Keywords:** alcoholic beverage, multiple regression analysis, consumer behavior

## Abstract

Alcoholic beverages held significant importance in global dietary cultures. Their consumption was subject to the combined influence of sociocultural, economic, and psychological factors. As one of the world’s major alcohol consumption markets, China exhibited increasingly diverse drinking patterns, yet research on drinking behaviors based on the Chinese population remained relatively limited. This study employed a questionnaire-based survey to collect data. A total of 2119 Chinese adult alcohol consumers were recruited between October 2024 and April 2025. The sample encompassed individuals with diverse demographic backgrounds, including variations in gender, age, education level, monthly income, and occupation. Based on this dataset, multivariate logistic regression analysis was applied to systematically examine the key factors influencing drinking frequency among Chinese adult drinkers. The study found that the majority of drinkers in China engaged in low- to moderate-frequency drinking, with significant variations observed across different demographic groups: women aged 31–50 showed a higher proportion of high-frequency drinking, while individuals over 50 experienced a notable decline in drinking frequency. Individuals with smoking habits and higher stress levels were more likely to engage in high-frequency drinking. In contrast, those who report higher subjective well-being tended to exhibit moderate-frequency drinking patterns, characterized by moderate but non-excessive consumption. This study constructed a multi-dimensional profile of alcohol consumption behavior in China, thereby providing precise guidance for future product positioning and development, promoting high-quality development in the alcoholic beverage industry, and offering a scientific basis for advocating a culture of moderate and healthy drinking.

## 1. Introduction

Alcoholic beverages serve as a distinctive symbol of human civilization and constitute an important component of global dietary culture [1]. Around the world, a rich variety of alcoholic beverages exists, with different types deeply influenced by natural conditions and historical cultural contexts. For example, the peat of the Scottish Highlands imparts a unique smoky flavour to single malt whisky [2], while the ancient Greek practice of sealing pottery jars with pine resin for wine storage eventually gave rise to a wine that represents Greek cultural identity today [3]. At the same time, the development and innovation of brewing technology are also key factors shaping the diverse flavour profiles of alcoholic beverages. The use of multiple distillations and activated carbon filtration in vodka production resulted in high purity [4]; the refreshing taste of lager beer was due to its low-temperature fermentation process [5], and Chinese baijiu exhibited its unique characteristics through solid-state fermentation and distillation techniques [6]. These technical differences in the brewing processes of various alcoholic beverages reflected humanity’s continuous exploration and pursuit of diverse flavours in alcoholic drinks.

Alcoholic beverages, owing to their entrenched cultural and social significance, maintain sustained high global demand [7]. According to Statista, the worldwide sales volume of alcoholic beverages reached approximately 2842.8886 billion liters in 2024 [8], with a total market value of RMB 14,371.482 billion. China accounted for RMB 3176.098 billion of this total, representing approximately one-fifth of the global market value. As one of the world’s largest alcoholic beverage markets, China displayed increasingly diversified consumer preferences. This landscape was characterized by a dual structure: baijiu, the national liquor, maintained its cultural significance in social and emotional communication [9], while younger demographics increasingly favored low-alcohol options like fruit beers and craft beers [10]. This trend was further reflected in the growing market presence of Western spirits such as whisky, which gained traction due to their complex flavor profiles. Underlying these shifts, rapid economic growth and consumption upgrades have been driving the market’s evolution from a focus on “quantity” to a pursuit of “quality.” On the production side, traditional alcoholic beverage enterprises utilized technologies such as intelligent sensing [11], microbiome omics regulation [12], and targeted extraction of flavor compounds [13] to optimize production processes, thereby increasing the output rate of premium products and achieving high-quality development. On the consumption side, the ultimate success and market recognition of this transformation fundamentally depended on consumer evaluation. It was established that the composition and concentration of key flavor compounds in beer dictated its dominant sensory attributes, such as sweetness and umami. These attributes were key determinants of consumer preference and acceptance [14]. Mandha et al. [15] emphasized the need to study and understand consumer perceptions and preferences to identify factors influencing consumer behavior and detect product parameters requiring further development. Thus, the market competitiveness derived from technological innovations was contingent upon their successful passage through consumers’ multidimensional evaluation framework. Research on the traits of Chinese drinking behavior was therefore critically important at this stage.

Research into the complex processes of food consumption and selection has yielded a variety of methodological approaches [16]. At a conceptual level, Moje [17] theorized a holistic framework that systematically delineated the influence of extrinsic and intrinsic product attributes, psychological states, situational contexts, and socio-cultural elements on food choices. Empirically, Chen [18] elucidated consumer sensory preferences for light beer by leveraging large-scale survey data and statistical modeling. In a health-focused study, Micek [19] analyzed questionnaire data via multivariate logistic regression, revealing a significant association between moderate wine consumption and a reduced risk of cognitive impairment. Other studies applied multilevel logistic regression models to examine the relationship between energy drink consumption and socio-demographic and behavioral factors [20]. However, most existing research derived from Western populations: The Armenian wine market, which prioritizes quality and word-of-mouth recommendations among relatives and friends [21], the well-structured Brazilian craft beer market that emphasizes flavor and packaging design [22], and the consumption of Hungarian homemade distilled spirits driven by emotional and national identity [23], all revealed behavioral patterns specific to their respective cultural contexts. Therefore, the sociocultural specificity of China limited the direct transferability of these research findings. Additionally, the operationalization of variables in the extant literature was relatively simplistic, with a predominant focus on demographic factors and a notable lack of depth in examining the underlying psychological drivers of consumption behavior.

Therefore, this study aimed to identify factors influencing the consumption behaviors of alcoholic beverage consumers, clarify the key drivers behind drinking consumption, and construct a multidimensional profile of Chinese alcoholic beverage consumers based on group differences, thereby providing an important foundation for the alcoholic beverage industry to adapt to market dynamics and formulate precise strategies. The research was conducted in the following parts: (1) A questionnaire survey was designed, consisting of two sections—basic information and personal circumstances—to investigate alcohol consumption among Chinese consumers, and the questionnaire data were collected. (2) Based on the survey results, a comparative analysis of drinking frequency across different demographic groups was conducted to clarify the characteristics of alcohol consumption behavior among various consumer categories. (3) Quantitative values were assigned to the responses in the personal circumstances section, and the scored data were analyzed using multivariate logistic regression to identify personal factors influencing the frequency of alcohol consumption among Chinese consumers.

## 2. Materials and Methods

### 2.1. Sample

This study utilized an online questionnaire administered via Questionnaire Star (Enterprise Standard Edition; Changsha Ranxing Information Technology Co., Ltd., Changsha, Hunan, China). A convenience sampling approach was adopted, with participants recruited nationwide primarily through online social media platforms such as WeChat. All respondents provided informed consent and participated voluntarily. Prior to full deployment, a preliminary survey was conducted with 20 individuals to assess question clarity and comprehensibility. Additionally, five internal experts were invited to review the questionnaire content. Their feedback was incorporated to optimize the structure and wording of the final survey. The questionnaire was distributed with Beijing as the primary base, extending across China and including overseas Chinese consumers. Data collection took place from October 2024 to April 2025. After excluding invalid responses, a total of 2119 valid questionnaires were retained for analysis. As an exploratory study relying on online convenience sampling, the sample was primarily composed of consumers within China, supplemented by overseas Chinese respondents to broaden perspective diversity. It is acknowledged that the sampling method limits the generalizability of the findings; thus, the results should be regarded as preliminary and require further validation through more extensive and representative samples in future research.

### 2.2. Measures

This study collected data on consumers’ basic information and personal circumstances through a questionnaire to analyze the characteristics of their alcohol consumption behaviors and influencing factors. Based on the questionnaire data, drinking behaviors across different demographic groups were compared, and multivariate logistic regression was employed to identify key factors influencing drinking frequency.

#### 2.2.1. Basic Information

The basic information section of the questionnaire included five questions regarding gender, age, income, education level, and occupation to collect key demographic details of the respondents, thereby providing a fundamental understanding of their background characteristics. The gender question offered three options: male, female, and non-binary. The age question included three options: 18–30 years old, 31–50 years old, and 50 years old and above. The income question provided seven options: 0–2000 RMB, 2001–4000 RMB, 4001–6000 RMB, 6001–10,000 RMB, 10,001–15,000 RMB, 15,001–30,000 RMB, and 30,001 RMB and above. The education level question included three options: junior high school and below, high school/secondary specialized education/associate degree, and bachelor’s degree and above.

#### 2.2.2. Personal Circumstances

The personal circumstances section included questions covering five aspects: social behavior, smoking behavior, cultural perception of alcohol, subjective well-being, and psychological stress. Alcohol consumption was not only a material consumption behavior but also a product of social contexts. McClain et al. [24] found a significant association between drinking behavior and social interactions, while other studies indicated that social settings such as parties, friends’ homes, and being surrounded by intoxicated persons increased the likelihood of heavy drinking [25]. Leila et al. [26] discovered that current smoking was associated with binge drinking, and smoking status could be used to identify and screen for problem drinkers, with this association being stronger among males and Asian/African populations. A multinational study by Pilatti et al. [27] demonstrated significant correlations between cultural orientations and both alcohol consumption and alcohol-related negative consequences. Existing research also suggested a U-shaped relationship between alcohol consumption and well-being [28]. Ueno et al. [29] found through their study that drinking habits were significantly associated with psychological stress responses.

The questionnaire offered two options for social behavior: social drinking and non-social drinking; two options for smoking behavior: smoking and non-smoking; two options for cultural perception of alcohol: possessing cultural awareness of alcohol and lacking cultural awareness of alcohol; two options for subjective well-being: happy and unhappy; and two options for psychological stress: higher stress and lower stress.

### 2.3. Data Analysis

This study utilized the Questionnaire Star platform for the distribution and collection of the survey questionnaires. The collected data were statistically analyzed using IBM SPSS Statistics (Version 25.0; IBM Corp., Armonk, NY, USA).

Logistic regression modeling represents one of the key methods for analyzing the relationship between a variable and its influencing factors. When the dependent variable has multiple categories, the following model (1) is recommended.

(1) $$\ln\left[\frac{p_{\left(y=j|x\right)}}{p_{\left(y=J|x\right)}}\right]=\alpha_{j}+\sum_{k}^{K}\beta_{kj}x_{kj}$$

As shown in Equation (1), assuming there were *J* categories of the dependent variable, ln [*p* (*y* = *j*|*x*)/*p* (*y* = *J*|*x*)] represented the natural logarithm of the odds of a specific event type j occurring relative to the reference category *J*. This model could be used to estimate the linear relationship between the dependent variable and its independent variables. Here, *x_kj_* denoted the independent variable for event type *j*, while *α_j_* and *β_kj_* represented the regression intercept and regression coefficient, respectively. The multinomial logit model established *J-1* independent models (one for each non-reference category *j*). Converting the regression coefficient *β_kj_* to Exp (*β_kj_*) indicated that when the corresponding independent variable *x_kj_* changed by one unit, the dependent variable ln [*p* (*y* = *j*|*x*)/*p* (*y* = *J*|*x*)] would change by a factor of Exp (*β_kj_*). Specifically, Exp (*β_kj_*) measured how many times the odds of event *j* occurring relative to the reference category *J* would change for each one-unit increase in the independent variable *x_kj_.*

To derive quantitative insights from the logistic regression, Average Marginal Effects (AMEs) were employed. AMEs measure the average change in the probability of the dependent variable resulting from a discrete change in an independent variable, providing a more interpretable measure of the predictors’ influence on the outcome.

### 2.4. Statistical Analysis and Statistical Methods

The questionnaire was administered online via Questionnaire Star (Enterprise Standard Edition; Changsha Ranxing Information Technology Co., Ltd., Changsha, Changsha, Hunan, China). Spearman’s correlation analysis were conducted using IBM SPSS Statistics (Version 25.0; IBM Corp., Armonk, NY, USA). The computation of Average Marginal Effects (AMEs) was conducted using Python (Version 3.13). Other statistical analyses and graphical visualizations were carried out in Origin 2024. A *p*-value < 0.05 was considered statistically significant.

## 3. Results

### 3.1. Sample Characteristics

**Figure 1:**
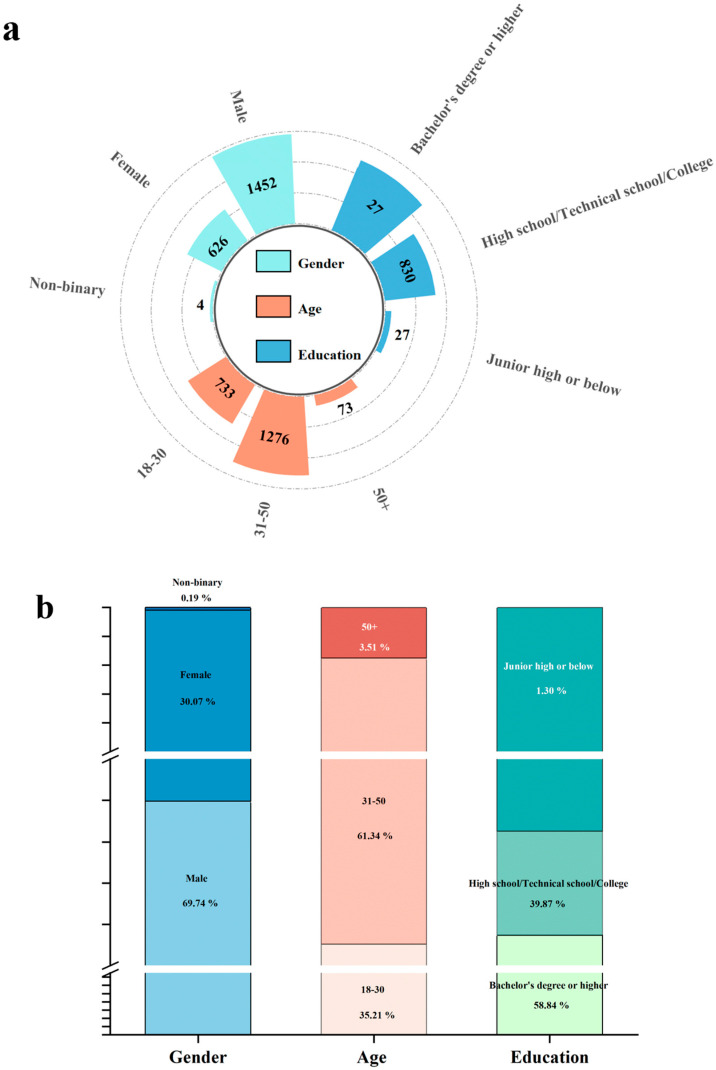
Frequency and proportion of consumers across categories. (**a**) Frequency distribution of different demographic groups. (**b**) Proportional distribution of different demographic groups.

To focus on the drinking population, non-drinkers were excluded from the demographic analysis. From the initial 2119 respondents, 2082 alcohol consumers were retained. As shown in Figure 1, the final sample consisted of 1452 males (69.74%) and 626 females (30.07%). The age distribution was as follows: 732 individuals (35.21%) were aged 18–30, while 1276 (61.28%) were 31–50, and 73 (3.51%) were over 50. In terms of education, 27 (1.30%) had a junior high school education or below, 830 (39.87%) had completed high school/vocational/associate education, and 1225 (58.84%) held a bachelor’s degree or higher. The sample thus provides a demographically diverse representation of adult alcohol consumers in China.

**Figure 2:**
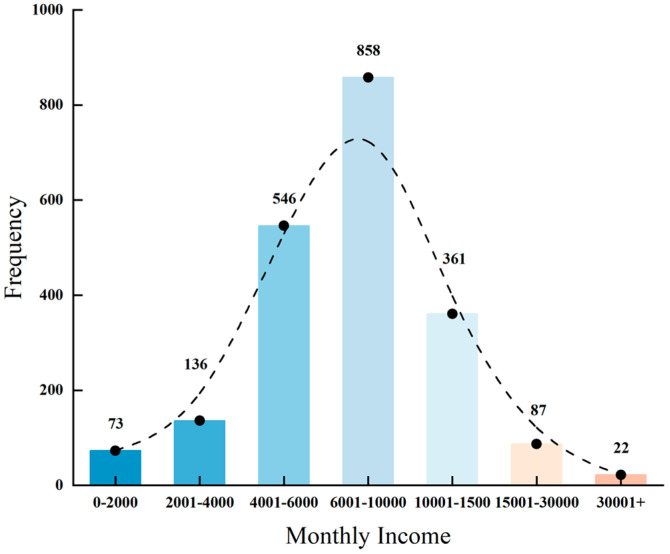
Frequency distribution of consumers across income brackets.

As shown in Figure 2, after excluding non-drinking respondents, the group with a monthly income of 6001–10,000 RMB was the largest, with a total of 858 people, accounting for 41.19%. This was followed by the group with a monthly income of 4001–6000 RMB, which included 546 people, representing 26.21%. Those earning 10,001–15,000 RMB per month numbered 361, accounting for 17.33%. The group with a monthly income of 15,001–30,000 RMB comprised 87 people, or 4.18% of the total. The proportion of individuals earning 2001–4000 RMB per month was relatively low, with 136 people, making up 6.53%. Those with a monthly income exceeding 30,000 RMB numbered 22, accounting for 1.06%. Meanwhile, the group with a monthly income of 0–2000 RMB included only 73 people, representing 3.50% of the sample. Overall, the distribution exhibited a normal distribution pattern dominated by the group with a monthly income of 6001–10,000 RMB.

**Figure 3:**
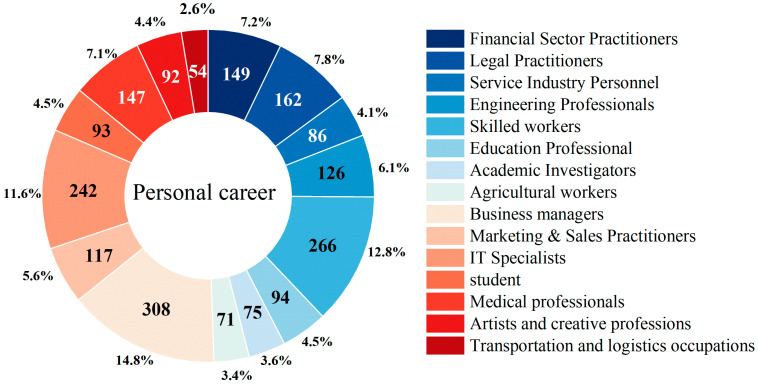
Frequency and Proportion of Consumers by Occupation.

As illustrated in Figure 3, the survey respondents spanned a wide spectrum of occupations. The occupational distribution was as follows: Business Managers accounted for the largest share (14.8%, *n* = 308), followed by Skilled Workers (12.8%, *n* = 266), IT Specialists (11.6%, *n* = 242), Legal Practitioners (7.8%, *n* = 162), Financial Sector Practitioners (7.2%, *n* = 149), Medical Professionals (7.1%, *n* = 147), Engineering Professionals (6.1%, *n* = 126), Marketing & Sales Practitioners (5.6%, *n* = 117), Education Professionals (4.5%, *n* = 94), Students (4.5%, *n* = 93), Artists and Creative Professionals (4.4%, *n* = 92), Service Industry Personnel (4.1%, *n* = 86), Academic Investigators (3.6%, *n* = 75), Agricultural Workers (3.4%, *n* = 71), and Transportation and Logistics Occupations (2.6%, *n* = 54).

### 3.2. Characteristics of Drinking Consumption Behavior of Different Groups of Consumers

In this study, the question regarding drinking frequency offered three response options: low-frequency (once per month or less), medium-frequency (2–8 times per month), and high-frequency (9 or more times per month). As shown in Table 1, the overall drinking frequency among respondents exhibited a gradually decreasing trend from low-frequency to high-frequency. Among them, 831 respondents who consumed alcohol selected the low-frequency drinking option, accounting for 39.91% of the total; 774 respondents selected the medium-frequency drinking option, representing 37.18%; and 477 respondents chose the high-frequency drinking option, making up 22.91%.

Drinking frequency varied across gender and age groups, as detailed in Table 1. Among male drinkers, 565 (38.91%) were low-frequency consumers, 550 (37.88%) moderate-frequency, and 337 (23.21%) high-frequency. Female drinkers showed a similar pattern, with the majority in the low-frequency category (265, 42.33%), followed by 222 (35.46%) moderate-frequency and 139 (22.20%) high-frequency consumers. Non-binary individuals were excluded from comparative analysis due to an extremely small sample size (fewer than 3 in each frequency category, each representing less than 0.5% of the total sample), which prevented meaningful statistical comparison. Age-based analysis revealed distinct consumption patterns. The 31–50 age group had the highest level of drinking participation, comprising 462 (36.21%) low-frequency, 505 (39.58%) moderate-frequency, and 309 (24.21%) high-frequency drinkers. The 18–30 age group also showed relatively high engagement, with 326 (44.47%) low-frequency, 255 (34.79%) moderate-frequency, and 152 (20.74%) high-frequency drinkers. In contrast, alcohol consumption declined substantially among those aged 50 and above, with only 43 (58.90%) low-frequency, 14 (19.18%) moderate-frequency, and 16 (21.92%) high-frequency drinkers, indicating a notable overall reduction in drinking activity within this older cohort.

**Figure 4:**
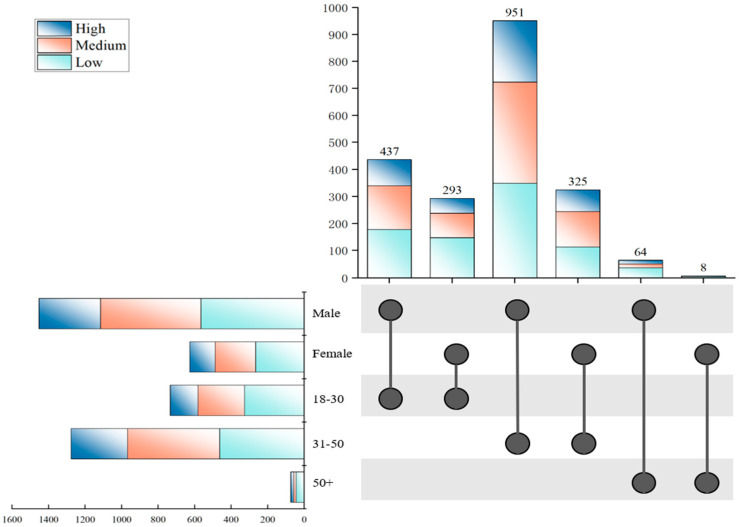
Frequency distribution of drinking patterns by consumer categories.

Due to the limited sample size of non-binary individuals (*n* = 4), this group was excluded from the drinking frequency analysis to prevent potential statistical bias. For clearer comparisons across demographic subgroups, the results are presented as proportional distributions of each drinking frequency category. Specifically, for each designated group (by gender and age), the number of individuals in each drinking frequency category was divided by the total number of people within that subgroup. As shown in Figure 4, after simultaneously considering gender and age factors, although males aged 31–50 constituted the largest subgroup in the total sample (951 individuals), their proportion of high-frequency drinkers was 23.97%, which is slightly lower than that of females in the same age group (24.92%). This finding somewhat challenged the traditional perception that “males generally have higher drinking frequencies than females.” Further analysis of the medium-frequency drinking group revealed that females aged 31–50 accounted for the highest proportion in this category, reaching 40.31%, indicating that drinking behavior had become relatively common and normalized among this demographic. Considering the characteristics of their life stage, women in this age group often face multiple pressures related to career development, children’s education, and family relationships. Alcohol consumption may serve as a convenient, private, and partially socially accepted method of emotional regulation. In contrast, low-frequency drinking was characterized by a strong representation of males aged 50 and above, who made up 59.38% of this category. This trend can be plausibly linked to heightened health awareness, age-related declines in alcohol tolerance, and increased focus on managing chronic conditions. As age advances, health considerations emerge as a primary factor leading to reduced drinking frequency. In terms of overall distribution, low- and medium-frequency patterns collectively represented the majority of respondents, with high-frequency drinkers constituting a distinct minority in every subgroup.

### 3.3. Exploration on the Link Between Personal Factors and Alcohol Consumption Behavior

#### 3.3.1. Descriptive Analysis of Personal Factors

**Figure 5:**
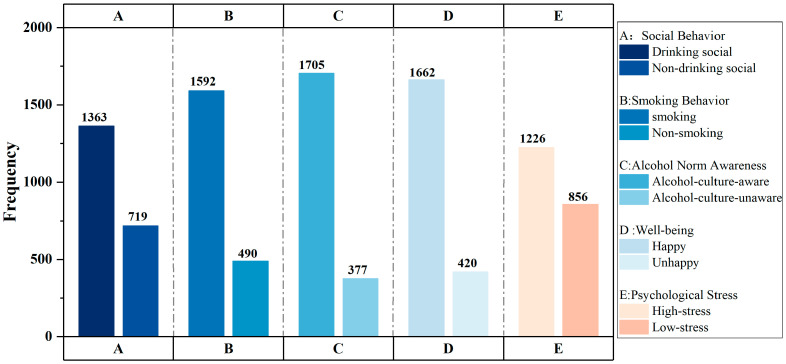
Distribution of personal situations of drinking consumers.

This survey analyzed the distribution characteristics of five key personal factors among 2082 alcohol-consuming consumers. As shown in Figure 5, the results revealed that in terms of social behavior, the majority of consumers (65.47%, *n* = 1363) exhibited a tendency toward social drinking, while 34.53% (*n* = 719) were non-social drinkers. Regarding smoking behavior, smokers constituted a significantly higher proportion than non-smokers (76.46%, *n* = 1592 vs. 23.54%, *n* = 490). In terms of cultural perception of alcohol, 81.89% (*n* = 1705) of consumers possessed foundational knowledge of alcohol culture, while only 18.11% (*n* = 377) lacked relevant awareness. The subjective well-being assessment found that nearly 80% of respondents (79.83%, *n* = 1662) reported a positive psychological state, whereas 20.17% (*n* = 420) indicated a lack of well-being. In the psychological stress evaluation, 58.89% (*n* = 1226) of consumers self-reported experiencing high levels of stress, while the remaining 41.11% (*n* = 856) reported lower stress levels.

#### 3.3.2. Analysis of Correlations Between Basic Information and Personal Factors

**Figure 6:**
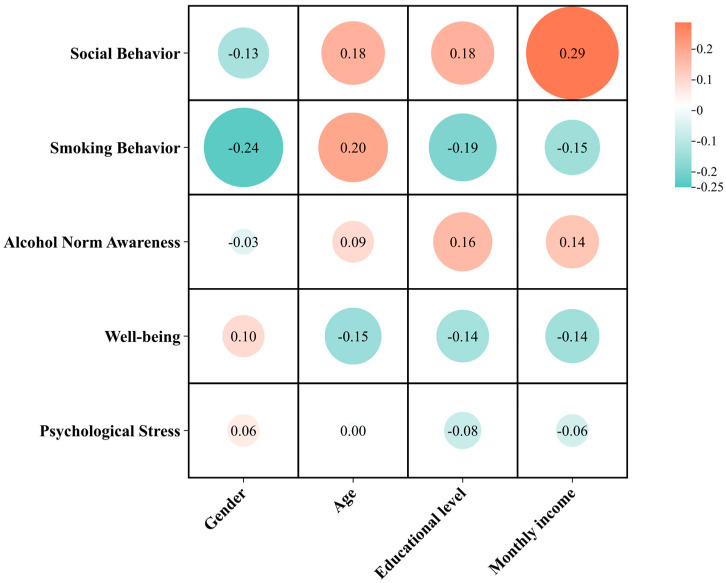
Correlation analysis between basic information and personal circumstances.

Based on 2082 valid samples, this study employed Spearman correlation analysis to explore the associations between demographic variables and personal factors. As shown in Figure 6, the statistical analysis revealed that gender showed a significant negative correlation with drinking socialization and smoking status (r = −0.13, *p* < 0.01; r = −0.24, *p* < 0.01), while it exhibited a significant positive correlation with well-being and psychological stress (r = 0.10, *p* < 0.01; r = 0.06, *p* < 0.01). The results indicated that gender differences significantly influenced behavior and psychological states: males engaged significantly more in drinking socialization and smoking than females. However, compared to women, men reported significantly lower subjective well-being yet also lower levels of psychological stress. This discrepancy may stem from gender differences in the perception and expression of stress, with men being more inclined to overlook their emotional symptoms. This reflects systematic gender-based differences in both social behavior and mental health.

The analysis revealed significant positive associations between age and drinking socialization (r = 0.18, *p* < 0.01), smoking status (r = 0.20, *p* < 0.01), and alcohol cultural awareness (r = 0.09, *p* < 0.01). Conversely, a significant negative association was observed between age and well-being (r = −0.15, *p* < 0.01). This indicates that the older demographic was not only characterized by greater engagement in drinking-related social and cultural practices but also by lower reported well-being.

Education level was significantly correlated with all five personal factors. It showed significant positive correlations with drinking socialization and alcohol cultural awareness (r = 0.18, *p* < 0.01; r = 0.16, *p* < 0.01) and significant negative correlations with smoking status, well-being, and psychological stress (r = −0.19, *p* < 0.01; r = −0.14, *p* < 0.01; r = −0.08, *p* < 0.01). Individuals with higher education levels and monthly income were more actively engaged in drinking socialization and recognized alcohol culture but had lower smoking rates. At the same time, they experienced greater psychological stress and lower well-being, indicating that those with higher socioeconomic status faced greater psychological challenges while having access to more social resources.

Monthly income level also exhibited significant correlations with personal factors. It showed significant positive correlations with drinking socialization and alcohol cultural awareness (r = 0.29, *p* < 0.01; r = 0.14, *p* < 0.01) and significant negative correlations with smoking status, well-being, and psychological stress (r = −0.15, *p* < 0.01; r = −0.14, *p* < 0.01; r = −0.06, *p* < 0.01).

#### 3.3.3. Analysis of Drinking Frequency Distribution Across Consumer Personal Factors

As shown in Table 2, among respondents who engaged in social drinking, low-frequency drinkers accounted for the highest proportion (557 individuals, 40.87%), followed by medium-frequency drinkers (494 individuals, 36.24%), while high-frequency drinkers represented the lowest proportion (312 individuals, 22.89%). Among respondents with smoking behavior, low-frequency drinkers constituted the highest proportion (604 individuals, 37.94%), followed by medium-frequency drinkers (593 individuals, 37.25%), with high-frequency drinkers accounting for the lowest proportion (395 individuals, 24.81%). Among respondents who possessed alcohol cultural awareness, low-frequency drinkers represented the highest proportion (704 individuals, 41.29%), followed by medium-frequency drinkers (634 individuals, 37.14%), while high-frequency drinkers accounted for the lowest proportion (367 individuals, 21.55%). Among respondents who reported subjective well-being (feeling happy), medium-frequency drinkers constituted the highest proportion (665 individuals, 40.01%), followed by low-frequency drinkers (644 individuals, 38.75%), with high-frequency drinkers representing the lowest proportion (353 individuals, 21.24%). Among respondents who self-reported high stress levels, medium-frequency drinkers accounted for the highest proportion (459 individuals, 37.44%), followed by low-frequency drinkers (404 individuals, 32.95%), while high-frequency drinkers represented the lowest proportion (363 individuals, 29.61%). In summary, across the five distinct personal factor groups, the distribution of drinking frequencies varied, with low- and medium-frequency drinkers dominating most groups.

Multicollinearity assessment was conducted for the five personal factors using Variance Inflation Factor (VIF). The results indicated that all VIF values were below 5 (ranging from 1.043 to 1.179), confirming the absence of multicollinearity. This allowed for subsequent regression analysis to be performed.

#### 3.3.4. Preliminary Analysis of Personal Factors on Drinking Frequency

**Figure 7:**
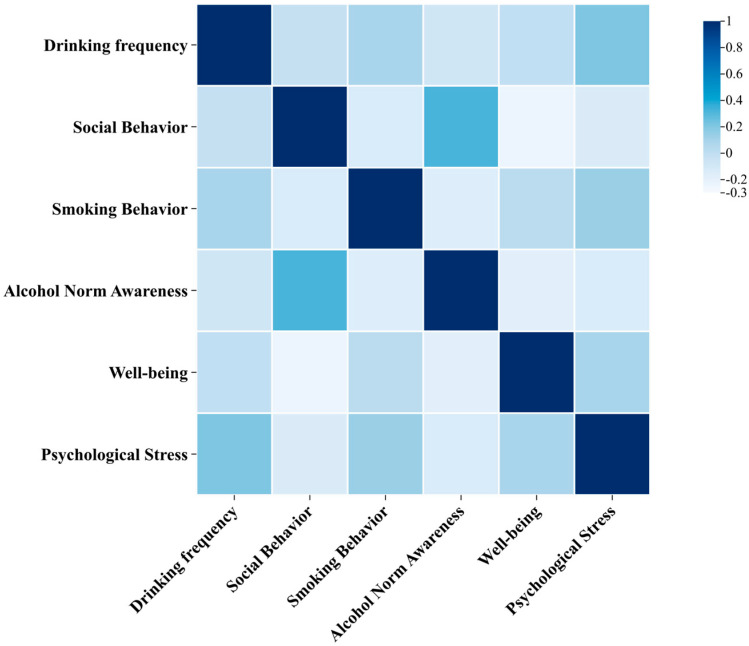
Correlation analysis between personal factors and drinking frequency.

This study first conducted a preliminary analysis of the associations between five personal factors and drinking frequency using Spearman correlation analysis, with results presented in Figure 7. Smoking behavior demonstrated a significant positive correlation with drinking frequency (r = 0.089, *p* < 0.001), indicating that smokers tended to drink more frequently than non-smokers. Similarly, alcohol cultural awareness was positively associated with drinking frequency (r = 0.075, *p* = 0.001), suggesting that respondents with greater knowledge of alcohol culture engaged in drinking more often. Psychological stress also showed a significant positive correlation with drinking frequency (r = 0.208, *p* < 0.001), implying that individuals reporting higher stress levels consumed alcohol more frequently. Although Spearman correlation analysis did not reveal statistically significant associations between social drinking behavior or subjective well-being and drinking frequency, existing literature indicates that these factors may influence drinking behavior through more complex pathways. For instance, Kehayes et al. [30] found that changes in social drinking motivation in one partner significantly predicted corresponding changes in the other partner’s social motivation through mediating pathways, which in turn positively predicted changes in weekly drinking frequency. Similarly, Chen et al. [31] discovered that among Chinese adolescents, well-being was negatively associated with drinking behavior, and this relationship was significantly enhanced when multiple unhealthy behaviors coexisted. The discrepancy between theoretical expectations and empirical findings may be attributed to the inherent limitations of Spearman correlation analysis, which can only capture monotonic relationships between two variables without controlling for potential confounders. To address this limitation and better understand the independent effects of multiple predictors, we subsequently employed multivariate logistic regression analysis to examine how these variables collectively influence drinking frequency.

### 3.4. The Influence of Personal Factors on the Frequency of Alcohol Consumption

This study examined the personal factors influencing alcohol consumption frequency. The dependent variable, drinking frequency, was coded as an ordinal categorical variable with three levels: low-frequency (coded as 1), medium-frequency (2), and high-frequency (3). Given the categorical nature of the outcome variable, a logistic regression approach was deemed appropriate. Furthermore, as the analysis involved five binary independent variables (personal factors), a multinomial logistic regression model was ultimately employed to assess their relationship with drinking frequency.

The likelihood ratio test for the model indicated overall significance (χ^2^ = 151.284, df = 10, *p* < 0.001), suggesting that the set of independent variables has a significant predictive effect on the categorization of drinking frequency. The goodness-of-fit measures for the model were as follows: McFadden’s pseudo R^2^ = 0.07, Nagelkerke’s pseudo R^2^ = 0.18. Although the pseudo R^2^ values are relatively low, this is common in studies investigating complex human behaviors.

The results of the multinomial logistic regression analysis are presented in Table 3. The analysis used low-frequency drinking as the reference group. The following conclusions were drawn:

$$\ln\left[\frac{p_{\left(y2\right)}}{p_{\left(y1\right)}}\right]=-0.802\;+\;0.527x_{4}\;+\;0.379x_{5}$$

$$\ln\left[\frac{p_{\left(y3\right)}}{p_{\left(y1\right)}}\right]=-1.126+0.417x_{2}+0.423x_{3}-0.376x_{4}+1.195x_{5}$$

Here, *y*_1_ represented low-frequency drinking, *y*_2_ represented medium-frequency drinking, and *y*_3_ represented high-frequency drinking; *x*_1_ denoted social behavior, *x*_2_ denoted smoking status, *x*_3_ denoted alcohol cultural awareness, *x*_4_ denoted subjective well-being, and *x*_5_ denoted psychological stress.

The regression analysis results indicated that among medium-frequency drinkers, two personal factors—subjective well-being and psychological stress—had a significant influence. Regarding subjective well-being, as the level of subjective well-being increased (i.e., the proportion of individuals feeling happy expanded relative to those feeling unhappy), the number of medium-frequency drinkers showed a significant upward trend. Enhanced well-being was positively associated with an increase in the medium-frequency drinking population (B = 0.527, *p* < 0.001), and a one-unit increase in x4 raised ln [p (y2)/p (y1)] by 0.527. This suggested that people were more likely to engage in medium-frequency drinking when they felt happier. Regarding psychological stress, when the proportion of individuals with high psychological stress increased (relative to those with low stress), the number of medium-frequency drinkers also rose significantly. Elevated psychological stress levels were positively correlated with the expansion of the medium-frequency drinking population (B = 0.379, *p* < 0.001), and a one-unit increase in x5 raised ln [p (y2)/p (y1)] by 0.379. This result indicated that people were more inclined to engage in medium-frequency drinking as their psychological stress levels increased. In summary, individuals with high subjective well-being and self-rated higher stress levels are more inclined to engage in moderate-frequency drinking.

The regression analysis results showed that high-frequency drinking behavior was significantly influenced by four personal factors: smoking behavior, alcohol cultural awareness, subjective well-being, and psychological stress. Regarding smoking behavior, as the proportion of smokers increased (compared to non-smokers), the proportion of high-frequency drinkers rose significantly. Smoking was positively correlated with the expansion of the high-frequency drinking population (B = 0.417, *p* = 0.006), and a one-unit increase in x2 raised ln [p (y3)/p (y1)] by 0.417. This indicated that individuals who smoked were more likely to engage in high-frequency drinking. In terms of alcohol cultural awareness, as the proportion of individuals with alcohol cultural awareness expanded (compared to those without such awareness), the number of high-frequency drinkers increased significantly. Enhanced alcohol cultural awareness contributed to a higher proportion of high-frequency drinkers (B = 0.423, *p* = 0.009), and a one-unit increase in x3 raised ln [p (y3)/p (y1)] by 0.423. This suggested that individuals with alcohol cultural awareness were more likely to engage in high-frequency drinking. Regarding subjective well-being, when the proportion of individuals feeling happy increased relative to those feeling unhappy, the proportion of high-frequency drinkers decreased significantly. That is, an increase in subjective well-being was negatively associated with the expansion of the high-frequency drinking population (B = −0.376, *p* = 0.009), and a one-unit increase in x4 reduced ln [p (y3)/p (y1)] by 0.376. This indicated that individuals who subjectively felt unhappy were more likely to engage in high-frequency drinking. In terms of psychological stress, as psychological stress levels rose, the proportion of high-frequency drinkers increased significantly. That is, elevated psychological stress levels promoted the expansion of the high-frequency drinking population (B = 1.195, *p* < 0.001), and a one-unit increase in x5 raised ln [p (y3)/p (y1)] by 1.195. This suggested that individuals with high stress levels were more likely to engage in high-frequency drinking behavior. In summary, individuals who smoke, possess alcohol cultural awareness, experience higher stress, and report lower subjective well-being are more likely to engage in high-frequency drinking.

Results from the multivariate logistic regression revealed that individuals who smoked, possessed alcohol cultural awareness, or experienced elevated stress levels were more likely to be high-frequency drinkers. In contrast, those reporting higher subjective well-being showed a greater propensity for moderate-frequency drinking—a pattern reflecting more regular consumption that remains controlled and non-abusive. Overall, these findings suggest that while certain factors such as smoking and stress drive a shift toward high-frequency drinking, well-being fosters a more regulated and moderate consumption profile.

To deepen the interpretation of the model, Average Marginal Effects (AMEs) were calculated to translate the regression coefficients into intuitive probability changes. As shown in the Appendix A, the AME analysis revealed that a high-stress state was the strongest predictor of high-frequency drinking, which significantly increased an individual’s probability of engaging in it by an average of 11.8 percentage points (*p* < 0.001). Furthermore, smoking increased the probability of high-frequency drinking by an average of 4.2 percentage points (*p* = 0.043). Notably, happiness exhibited a protective effect, reducing the probability of high-frequency drinking by an average of 3.5 percentage points (*p* = 0.062).

The AME results elucidated the distinct factors influencing drinking patterns: high stress and smoking habits significantly elevated the probability of high-frequency alcohol consumption. In contrast, happiness served as a protective factor that reduced the incidence of high-frequency drinking. These AME findings not only corroborated the conclusions of the multivariate logistic regression but also enhanced the model’s explanatory power by moving from statistical significance to practical relevance, thereby demonstrating its excellent goodness-of-fit.

## 4. Discussion

### 4.1. Drinking Frequency Across Different Personal Profiles

The drinking population exhibited a generally lower frequency of alcohol consumption among women, a pattern that was attributable to several factors. These included women’s typically lower alcohol tolerance [32], an increasingly strong commitment to healthier lifestyles [33,34], and a lower propensity to frequent drinking establishments such as bars or breweries [35]. Despite this trend, Carvalho et al. [34] highlighted that evolving consumption habits and a rising interest in market products were progressively establishing women as a crucial target demographic for the alcoholic beverage industry. This trend is particularly evident among female consumers aged 31–50: the proportion of moderate- and high-frequency drinkers in this group is not only higher than that of women in other age groups but also significantly exceeds that of their male counterparts. This finding contrasts sharply with the conventional notion that “men drink more than women,” suggesting a shift in gender roles within alcohol consumption and providing a new theoretical perspective and basis for dialogue in related research fields.

This study identified significant differences in drinking behaviors across age groups. Adults aged 18 to 50, particularly the 31–50 subgroup, exhibited the highest drinking frequency, which may be attributed to their more active social lifestyles. In contrast, a substantial reduction in alcohol consumption was observed after age 50, likely influenced by growing health awareness and age-related physiological changes. Notably, despite these age-related disparities, low and medium-frequency drinking emerged as the dominant pattern across all cohorts. This consistency suggests that moderate drinking has been successfully promoted and internalized as a deeply ingrained social norm. This finding resonates with the study by Thomas et al. [36], which reported a public preference for the ideal of “merriment not wasted,” consciously distinguishing it from unrestrained binge drinking culture. Together, this evidence points to a broader societal shift toward more civilized and temperate drinking practices.

### 4.2. Personal Factors Associated with Drinking Frequency

A statistically significant positive correlation was observed between smoking status and drinking frequency. Individuals who smoked engaged in high-frequency drinking significantly more often than non-smokers. A statistically significant positive correlation was observed between psychological stress and drinking frequency. As psychological stress levels increased, both medium- and high-frequency drinking populations showed significant expansion trends.

A statistically significant correlation existed between subjective well-being and drinking frequency. Compared to those who reported feeling unhappy, individuals who reported happiness more frequently engaged in medium-frequency drinking and were less inclined toward high-frequency drinking. This may stem from higher self-regulatory capacity and lower emotional drinking motives [34,35]. Nevertheless, the causal pathway between these factors requires verification in future research.

An individual’s level of cultural awareness regarding alcohol influenced their drinking frequency. Compared to those lacking cultural awareness of alcohol, groups possessing such awareness demonstrated a significantly higher probability of high-frequency drinking. A potential explanation for this phenomenon is that deep-seated cultural traditions amplify the role of alcohol consumption in expressing identity and reinforcing social belonging. However, the underlying causal mechanisms still require verification in future studies.

The results of this study indicate that individuals who smoke and experience elevated stress levels are more likely to engage in high-frequency drinking behavior. In contrast, those with higher subjective well-being tend to exhibit moderate-frequency drinking patterns—while they may drink more frequently, they maintain self-regulation and do not develop alcohol abuse. This finding not only confirms the promotive effect of risk factors on drinking behavior but also reveals that well-being may act as a protective factor, helping to keep alcohol consumption within moderate limits. These insights challenge the conventional perspective that views drinking behavior solely from a negative standpoint and emphasize the need to consider the dual roles of both risk and protective factors.

The results of this study showed no statistically significant association between social behavior and drinking frequency, although previous literature had indicated a positive correlation. For example, Park et al. [37] found that social activities (such as “social obligations requiring drinking”) significantly increased individual drinking frequency through cognitive mechanisms. This inconsistency might have arisen because this study focused on social behaviors primarily occurring in specific social environments centered around alcohol consumption, whereas previous literature examined a broader range of social activities, including many non-alcohol-related social scenarios. Therefore, the relationship between social behavior and drinking frequency requires evaluation considering contextual specificity.

This study is explicitly exploratory in nature, employing methods such as convenience sampling and single-dimension measurements for preliminary investigation. We fully acknowledge that the generalizability of the findings is limited by sample representativeness and measurement instruments. This study exploratorily revealed the relationship between personal characteristics and alcohol consumption behavior of Chinese consumers. However, it must be acknowledged that the use of binary questions to assess happiness and stress constituted a limitation of this research, which restricted our investigation into their continuous spectrum. Therefore, these findings urgently require future studies to validate and expand upon them through standardized multi-item scales. Nevertheless, these initial results provide valuable foundational evidence for the field and suggest directions for further in-depth research.

### 4.3. Research Contributions and Future Directions

Based on the research findings, the alcoholic beverage industry may consider the following strategic directions: develop differentiated products tailored to the characteristics of different consumer groups, such as seeking a balance between flavor innovation and health-oriented offerings. Simultaneously, the industry should collectively promote the concept of “moderate drinking,” which can be facilitated through transparent packaging information and package size designs that guide rational consumption. Furthermore, incorporating Chinese cultural elements into product design can enhance cultural identity and product value. These directions provide potential pathways for the industry to adapt to market changes; however, their specific implementation effects require further validation through market practice.

When contextualized within the broader international and cross-beverage literature, the Chinese drinking consumption demonstrates distinct characteristics. These stand in contrast to the food-pairing and regional heritage emphasized in European wine culture [38], the pursuit of self-expression and flavor exploration in the North American craft beer market [39], and the formal ritualism and generational traditions found in Japanese sake culture. This not only deepens the understanding of global drinking diversity but also provides a more nuanced theoretical foundation for formulating effective public health policies tailored to specific cultural contexts.

## 5. Conclusions

This study systematically revealed the key characteristics and primary influencing factors of the adult drinking population in China. The findings indicated that the overall drinking frequency among Chinese adults was predominantly low to medium, with “moderate drinking” gradually emerging as a widely accepted health concept.

In terms of population differences, women generally exhibited lower overall drinking frequency, but the proportion of medium-to-high frequency drinkers was significantly higher among those aged 31–50, identifying them as a crucial target demographic for the alcoholic beverage market. Age-stratified analysis showed that the 18–50 age group engaged in drinking more frequently, while individuals over 50 significantly reduced their alcohol consumption, reflecting the influence of life cycle on drinking patterns.

Further analysis based on multivariate logistic regression combined with Average Marginal Effects (AMEs) demonstrated that smoking habits and psychological stress were significant promoting factors for high-frequency drinking behavior. Conversely, higher subjective well-being was closely associated with moderate and stable medium-frequency drinking patterns, suggesting a positive regulatory role of mental health in drinking behavior.

This study not only provided an empirical basis for market segmentation positioning and product strategies in the alcoholic beverage industry but also offered directional guidance for its transition toward high-quality and sustainable development. Additionally, the research findings delivered robust data support for advocating and promoting a culture of rational drinking, carrying significant public health implications and societal value.

## Figures and Tables

**Table 1:** Frequency table of drinking patterns by consumer categories.

		Alcohol Consumption Frequency		
		Low	Medium	High
Gender	Male	565 (38.91%)	550 (37.88%)	337 (23.21%)
	Female	265 (42.33%)	222 (35.46%)	139 (22.20%)
	Non-binary	1 (25.00%)	2 (50.00%)	1 (25.00%)
Age	18–30	326 (44.47%)	255 (34.79%)	152 (20.74%)
	31–50	462 (36.21%)	505 (39.58%)	309 (24.21%)
	50+	43 (58.90%)	14 (19.18%)	16 (21.92%)
Total (*n*)		831 (39.91%)	774 (37.18%)	477 (22.91%)

Note: Figures denote frequency counts and row-wise percentages (*n* %). Percentages represent the distribution of responses within each subgroup (e.g., within all males) and are calculated horizontally.

**Table 2:** Frequency and percentage of drinking frequency among Consumers with different personal factors.

	Low	Medium	High	VIF
Drinking social	557 (40.87%)	494 (36.24%)	312 (22.89%)	1.179
Non-drinking social	274 (38.11%)	280 (38.94%)	165 (22.95%)	
smoking	604 (37.94%)	593 (37.25%)	395 (24.81%)	1.047
Non-smoking	227 (46.33%)	181 (36.94%)	82 (16.73%)	
Alcohol-culture-aware	704 (41.29%)	634 (37.14%)	367 (21.52%)	1.154
Alcohol-culture-unaware	127 (33.69%)	140 (37.14%)	110 (29.18%)	
Happy	644 (38.75%)	665 (40.01%)	353 (21.24%)	1.076
Unhappy	187 (44.52%)	109 (25.95%)	124 (29.52%)	
High-stress	404 (32.95%)	459 (37.44%)	363 (29.61%)	1.043
Low-stress	427 (49.88%)	315 (36.80%)	114 (13.32%)	

Note: Figures denote frequency counts and row-wise percentages (*n* %). Percentages represent the distribution of responses within each subgroup (e.g., within all individuals who engage in social drinking) and are calculated horizontally.

**Table 3:** Results of Multivariate Logistic Regression Analysis.

						95% CI for (Exp(B))	
Alcohol Consumption Frequency		B	Wald X^2^	*p*	Exp(B)	Lower	Upper
Medium		−0.802	12.578	<0.001			
	Drinking social	0.039	0.116	0.733	1.040	0.831	1.301
	Non-drinking social	0 ^b^					
	smoking	0.144	1.480	0.224	1.155	0.916	1.458
	Non-smoking	0 ^b^					
	Alcohol-culture-aware	0.046	0.102	0.749	1.047	0.790	1.389
	Alcohol-culture-unaware	0 ^b^					
	Happy	0.527	14.698	**<** **0.001**	1.693	1.294	2.217
	Unhappy	0 ^b^					
	High-stress	0.379	13.442	**<** **0.001**	1.460	1.193	1.788
	Low-stress	0 ^b^					
High		−1.126	18.602	<0.001			
	Drinking social	0.178	1.692	0.193	1.195	0.914	1.562
	Non-drinking social	0 ^b^					
	smoking	0.417	7.671	**0.006**	1.517	1.130	2.037
	Non-smoking	0 ^b^					
	Alcohol-culture-aware	0.423	6.894	**0.009**	1.526	1.113	2.092
	Alcohol-culture-unaware	0 ^b^					
	Happy	−0.376	6.752	**0.009**	0.687	0.517	0.912
	Unhappy	0 ^b^					
	High-stress	1.195	83.287	**<** **0.001**	3.303	2.556	4.270
	Low-stress	0 ^b^					

Note: The reference category for alcohol consumption frequency was the low-frequency drinking group. A value of “0 ^b^” for each variable indicates the reference group within that subgroup.

## Data Availability

The original contributions presented in the study are included in the article, further inquiries can be directed to the corresponding author.

## Appendix A

**Table A1:** Average Marginal Effect (AME) Results.

		AMEs	SE	95% CI		*p*
Medium	Drinking social vs. Non-drinking social	0.008	0.024	−0.038	0.054	0.734
	Smoking vs. Non-smoking	0.015	0.025	−0.033	0.063	0.543
	Alcohol culture vs. None	0.005	0.030	−0.053	0.063	0.867
	Happy vs. Unhappy	0.052	0.028	−0.002	0.106	0.062
	Low-stress vs. High-stress	0.038	0.022	−0.004	0.080	0.080
High	Drinking social vs. Non-drinking social	0.012	0.019	−0.024	0.048	0.518
	Smoking vs. Non-smoking	0.042	0.021	0.001	0.083	**0.043**
	Alcohol culture vs. None	0.041	0.023	−0.004	0.086	0.072
	Happy vs. Unhappy	−0.035	0.019	−0.072	0.002	0.062
	Low-stress vs. High-stress	0.118	0.025	0.069	0.167	**<** **0.001**
